# *Fraxinus xanthoxyloides* leaves reduced the level of inflammatory mediators during in vitro and in vivo studies

**DOI:** 10.1186/s12906-016-1189-7

**Published:** 2016-07-19

**Authors:** Tahira Younis, Muhammad Rashid Khan, Moniba Sajid, Muhammad Majid, Zartash Zahra, Naseer Ali Shah

**Affiliations:** Department of Biochemistry, Faculty of Biological Sciences, Quaid-i-Azam University, Islamabad, 45320 Pakistan; Department of Biosciences, COMSATS Institute of Information Technology, Islamabad, Pakistan

**Keywords:** Anti-inflammatory, Analgesic, *Fraxinus xanthoxyloides*, NFkB, Nitric oxide, TNF-α

## Abstract

**Background:**

Different parts of *Fraxinus xanthoxyloides* Wall. (Oleaceae) are used traditionally in the treatment of internal wounds, bone fracture, pain, jaundice, malaria and in pneumonia. These ailments involve protective and essential mechanisms of the organism in response to infection, injury and trauma. However, prolonged inflammation may lead to inflammatory disorders. The present investigation was carried to evaluate the crude methanol extract of *F. xanthoxyloides* leaves and its fractions for their anti-inflammatory and analgesic effects.

**Methods:**

Methanol extract of *F. xanthoxyloides* leaves was fractionated through liquid-liquid partition on escalating polarity of solvents. Acetic acid and thermal responses were used to evaluate the analgesic effects of extract/fractions in rat. Anti-inflammatory effects were monitored through in vitro; TNF-α activated NFkB in 293/NFkB-Luc HEK cells and LPS-activated nitric oxide (NO) assay in RAW 264.7 cells. For in vivo studies carrageenan induced paw edema model was used in rat. Both in vitro and in vivo studies have indicated that chloroform fraction exhibited superior anti-inflammatory effects to other extract/fractions and therefore, was used in air pouch model in rat to estimate the inhibition in leukocyte migration and synthesis of inflammatory mediators. In addition, phytochemical investigation of crude extract was carried out by GC-MS analysis.

**Results:**

GC-MS studies of crude extract revealed the presence of various classes of which terpenoids (26.61 %), lactam (16.47 %), esters (15.81 %), phenols (8.37 %), and steroid (6.91 %) constituted the major categories. Among the extracts chloroform fraction (200 mg/kg bw) significantly (*P* <0.001) increased the percent latency time (76.13 ± 4.49 %) in hot plate test after 120 min and decreased (*P* <0.001) the count of writhes (77.23 ± 5.64 %) as compared to other extracts. The in vitro studies indicated that chloroform fraction at 15 μg/ml more effectively inhibited the TNF-α induced synthesis of NFkB (85.0 ± 8.12 %, IC_50_ = 5.98 μg/ml) and LPS-instigated nitric oxide (78.23 ± 2.39 %, IC_50_ = 6.59 μg/ml) synthesis. Although all the extract/fractions showed a dose dependent increase in inhibition of edema formation however, chloroform fraction (4^th^ h = 77.64 ± 3.04 %) at 200 mg/kg bw exhibited relatively higher (*P* <0.001) anti-inflammatory activity in carrageenan-induced paw edema in rat. Moreover, chloroform fraction had the ability to decrease (*P* <0.001) the influx of leukocytes and the concentration of inflammatory mediators; TNF-α, NO, IL-6 and PGE_2_ in air pouch exudate.

**Conclusion:**

The study demonstrates the therapeutic potential of *F. xanthoxyloides* leaves against the inflammatory disorders suggesting the presence of active constituents in chloroform fraction.

## Background

Inflammation usually occurs nearly in all diseases and is considered to be an important causative agent for mortality and morbidity. Inflammation usually involves several metabolic and cellular events coordinated by mediators like cytokines, interleukins, prostaglandins and thromboxanes. A number of disorders such as rheumatoid arthritis, atherosclerosis and asthma have high prevalence worldwide. A number of studies have demonstrated that inflammation is key player that incite or sustain these disorders [1]. There is an up-regulation of tumor necrosis factor (TNF), interferon (INF)-γ and interleukin (IL)-1, IL-6, IL-12, IL-18 in early phase of inflammation, which stimulate recruitment of additional neutrophils and macrophages that are associated with the enhanced synthesis of inflammatory mediators such as nitric oxide (NO) and prostaglandin (PGE_2_) [2]. Additionally inflammatory responses augment the leukocytes, macrophages and mast cells to undergo various cellular changes of which the most important are the phagocytic uptake, mast cell degranulation [3] and generation of reactive oxygen species (ROS) [4–6]. These events ultimately incite or endure inflammatory responses together with induction of lipid peroxidation of membranous system and release of damaging macromolecules [4, 6]. Enhanced production of ROS up-regulate the synthesis of nuclear factor kappa B (NFkB) which in association with other mediators increase the generation of enzymes such as cyclooxygenase (COX)-2 and inducible nitric oxide synthase (iNOS). It is suggested that NFkB also regulate the anti-apoptotic responses [4]. As an antagonist to pathophysiological response of living tissues an increase has been recorded in release of interleukins (IL-4, IL-10, IL-13) and INF-α which together act as anti-inflammatory agents [7]. Finally extravasation of fluids and infiltration of leukocytes at the inflammatory area promote the edema formation [8]. Prolong sustainability of inflammation might result in many inflammatory disorders [9].

These inflammatory responses usually culminate in swelling, warmth, redness, pain and dysfunction. Such type of harmful stimuli are sensitized by specific type of neurons called as nociceptors which are embedded in the epithelium of skin, blood vessels, joints and many internal vital organs. These neurons may be mylenated or not and are activated by heat, cold or an injury and transmit these signals to the central nervous system. Increased secretion of cytokines either from leukocytes and/or central nervous system stimulates the peripheral nociceptors [10].

In spite of the recorded adverse effects of non-steroidal anti-inflammatory drugs (NSAIDs) are the clinicians’ first choice throughout the world to relieve pain and inflammation [11, 12]. Mechanistically they are known to relieve pain and inflammation by decreasing the levels of prostaglandins (PGs) like PGE2 and PGI2 and inhibiting the cyclooxygenase (COX) enzymes [13, 14]. Use of PGE2 in inflammation provides moderate level of protection to the gastrointestinal (GI) mucosa by increasing the release of mucus and bicarbonates. However, continuous use of such therapeutics might result in ulceration of lumen and bleeding of intestinal mucosa which collectively known as NSAIDs induced enteropathy. The major factor which contributes to the toxicity of GI is the inhibition of COX but there is also the contribution of other factors like endoplasmic reticulum stress and inflammation [15, 16]. These may be the effects triggered directly by NSAIDs or appear as downstream effects of COX inhibition. To minimize the adverse effects of NSAIDs, SC-560 as COX-1 selective inhibitor and celecoxib as selective inhibitor of COX2 were developed with the assurance that they will cause less GI toxicity than nonselective NSAIDs [17]. But still these agents employ stomach acidity, bleeding, ulceration [18, 19] and hepatotoxicity (elevated levels of serum alanine aminotransferase and aspartate aminotransferase), renal toxicity (increased creatinine level, peripheral edema and hypertension) and cardiovascular toxicity (angina, myocardial infarction, venous blockage, stroke) have been recorded in patients with use of relatively high doses of these compounds [20–22]. NSAIDs are also known to cause periodic colonic ulcers. The use of other NSAIDs like nicorandil is associated with anal and oral ulceration and many patients develop anaemia, diarrhoea or weight loss. However, the mechanism is yet to be established [23].

The main risk factors in ulcers related to the drugs are dependent on the dose and choice of NSAIDs and co-prescription of warfarin or corticosteroids. The risk of complications in the ulcer increases with increase in dose. Several meta-analyses have recommended a hierarchy of risk from a low level with low dose of ibuprofen, through the transitional level with diclofenac and to a high level with indomethacin, naproxen, piroxicam, ketoprofen [24]. NSAIDs injure the upper and lower gut and mucosa by depleting the COX-1 derived prostaglandins [25].

On account of low cost and less side effects plant based medicines are preferred worldwide as an alternative of NSAIDs because of the active anti-inflammatory phyto-constituents. There is an increasing trend in the use of plant based medicines and nearly 80 % of the world population prefers these drugs [26]. Nowadays drug discovery from the plants is based upon the bioactive guided fractionation and isolation which has resulted in development of many important drugs [26]. The first drug from plant source which was introduced in the market in 1826 was morphine and the first semi-synthetic pure drug was aspirin which was introduced in 1899 by Bayer is based upon a natural product salicin isolated from *Salix alba*. After that there was isolation of many other plant based drugs like digitoxin, pilocarpine, cocaine, quinine, codeine some of which are still in use [27]. Plant derived drugs which have been introduced in the market during the last few decades include Artemisinin from *Artemisia annua* to fight with multidrug resistant malaria, Paclitaxel isolated from *Taxus brevifolia* for ovarian, breast and lung cancer, Silymarin from *Silybum marianum* used for the treatment of liver diseases [28]. A semisynthetic compound Apomorphine which is used for the treatment of Parkinson’s disease is a derivative of morphine (*Papaver somniferum*). Tiotropium derived from atropine (*Atropa belladonna*) is used in chronic obstructive pulmonary disease. From *Cannabis sativa*, Dronabinol and Cannabidiol and Capsaicin from *Capsicum annuum* were obtained and are used as pain relievers. From *Catharanthus roseus*, Vincristine and vinblastine were extracted which are highly treasured anticancer drugs. Galantamine acquired from *Galanthus nivalis* is a natural alkaloid for Alzhemer’s are further prominent examples of secondary plant metabolites [29]. Other very important plant based anti-inflammatory compounds i.e. curcumin, colchicine, resveratrol, epigallocatechin-3-gallate (EGCG) and quercetin are in clinical trial [30].

*Fraxinus xanthoxyloides* (Wall. ex G.Don) DC. (Family Oleaceae) commonly known as Afghan ash is found in Northern areas of Pakistan, Afghanistan, India, Morocco and in Algeria. In northern areas of Pakistan its leaves and root bark has been used to treat jaundice, malaria and pneumonia. Decoction of stem bark (2–3 g) is given to reduce pain during labor [31]. Its wood is reported to be used in bone fracture [32]. Decoction of bark is used to expel pre-mature infant after death [33] and to cure internal wounds/injuries [34]. Decoction of stem/twigs is also used in wounds and bone fractures in cattle [35]. Remarkable anti-nociceptive and anti-inflammatory activities of the methanol extract at 200 and 400 mg/kg doses of *Fraxinus floribunda* leaves have been reported [36]. Administration of 10 mg/kg of methanol extract of the aerial parts of *F. micrantha* produced significant anti-inflammatory effects against carrageenan-induced acute inflammation in mice [37]. Xanthoxyloidin, a new biscoumarin together with esculetin, 5,7- dihydroxycoumarin and 6,8-dihydroxy-7-methoxycoumarin were isolated from the methanol extract of the whole plant of *F. xanthoxyloides* [38]. Intraperitoneal administration of stem bark extract of *F. ornus* displayed anti-inflammatory activity in both zymosan- and carrageenan-induced paw edema in mice [39]. Anti-nociceptive and anti-inflammatory activities of the methanol extract at 200 and 400 mg/kg doses of *F. floribunda* leaves have been reported [40]. There is no earlier report about the anti-inflammatory and analgesic activity of any extract or fractions of this plant. In this study we examined the anti-inflammatory and analgesic effect of the crude methanol extract of *F. xanthxyloides* leaves and its fractions. In this investigation we have evaluated the methanol extract of *F. xanthxyloides* stem bark and its derived fractions for anti-nociceptive and anti-inflammatory activities. GC-MS analysis of the crude extract was also performed.

## Methods

### Plant collection

The plant of *F. xanthoxyloides* was collected and identified by Dr. Rizwana Aleem Qureshi, Professor at the Department of Plant Sciences, Quaid-i-Azam University, Islamabad. Leaves of the *F. xanthxyloides* were collected in October, 2013 from the campus of Quaid-i-Azam University Islamabad, and a voucher specimen (45679) was submitted to Herbarium of Pakistan, Quaid-i-Azam University Islamabad, Pakistan.

### Extract preparation

The leaves obtained were dried under shade at 25 ± 2.0 °C. Powder of *F. xanthoxyloides* leaves (1.5 kg) was extracted with 95 % methanol (4.5 L) twice for 72 h at 25 ± 2.0 °C. The filtrate obtained was mixed and dried under vacuum in a rotatory evaporator at 40 °C to get 150 g of the crude methanol extract (FXM). A portion (100 g) of FXM was suspended in 400 ml of distilled water and fractionated by liquid-liquid partition by the addition of 400 ml of each solvent twice in order of *n*-hexane, chloroform, ethyl acetate and *n*-butanol. The organic layers were separated and dried under vacuum in a rotatory evaporator at 40 °C. Before drying the layers of the respective solvent were mixed together. The yield obtained for each solvent was; 18.45 g for *n*-hexane (FXH), 12.0 g for chloroform (FXC), 9.01 g for ethyl acetate (FXE), 12.83 g for *n*-butanol (FXB) and the soluble aqueous fraction (FXA) was 43.33 g. Crude extract and fractions were stored at 4 °C for various assays.

### Animals

We have used the Sprague-Dawley rats (150–200 g) of either sex for various studies. The animals were maintained in the primate facility at Faculty of Biological Sciences, Quaid-i-Azam University Islamabad, Pakistan at standard condition of temperature (25 ± 1 °C) and 12/12 h light/dark cycle. The animals have free access to feed and water *ad libitum*. The use of animals for experimentation was conducted according to the guidelines of National Institute of Health (NIH), Islamabad, Pakistan. The protocol followed in this study was approved (Bch#0263) by the Ethical committee of Quaid-i-Azam University Islamabad, Pakistan.

### Acute toxicity studies

To determine the acute toxicity of the extract/fractions, the guidelines 425 advocated by the Organization for Economic Cooperation and Development (OECD) were followed [41]. For this purpose Sprague-Dawley rats (*n* = 6) including males and females were orally administered at various doses of the extract/fractions (50, 250, 500, 1000, 2000, 3000 mg/kg) par oral (p.o.), whereas saline (10 ml/kg) was administered to the control rats. The animals were examined once daily for 14 days for mortality, behavioral pattern (lethargy, sleep, salivation), changes in physical appearance, injury, pain, and signs of illness.

## Pharmacological activities

### Analgesic studies

#### Acetic acid induced writhing test

To determine the analgesic activity of the extract/fractions obtained from the leaves of *F. xanthoxyloides*, a method previously reported by Khan et al. [42] was employed. In this model diclofenac sodium and aspirin were used at a concentration of 10 mg/kg, par oral (p.o) as standard drugs. Total number of writhing movements following p.o. administration of acetic acid solution (10 ml/kg, 1 %) was recorded over a period of 10 min, starting 5 min after acetic acid injection. Rats (6 in each group) were treated with FXM and its derived fractions; FXH, FXC, FXE, FXB and FXA (100 and 200 mg/kg), vehicle (saline), standard drugs (diclofenac sodium and aspirin 10 mg/kg), 30 min before acetic acid injection. The numbers of writhing movements (Constriction of abdominal muscles along with the stretching of hind limbs) were counted in both untreated and treatment groups and percentage inhibition in abdominal writhing was calculated as follow.

$$ \%\ \mathrm{inhibition}\ \mathrm{of}\ \mathrm{abdominal}\ \mathrm{writhing}=\left[\frac{\mathrm{Wc}\hbox{--} \mathrm{W}\mathrm{t}\Big)\ }{\left(\mathrm{W}\mathrm{c}\right)} \times 100\right] $$

W = No. of writhing, c = Control, and t = Test.

### Hot plate test

Sprague-Dawley rats of either sex weighing 150–200 g were used in this experiment to perform the analgesic test [43]. Before the animals were used to determine the analgesic effect of various extract/fractions; animals were pre-tested on hot plate analgesimeter (Havard apparatus Ltd., UK) which was maintained at 55 ± 0.1 °C to exclude the rats exhibiting >15 s of latency period. Animals were divided randomly into 15 groups with six rats in each group. In this study the animals of negative control were injected with normal saline 2 ml, i.p. whereas the animals of the positive groups received 10 mg/kg, p.o. of the diclofenac sodium and morphine respectively. Animals of the other groups were administered with doses of 100 mg/kg, p.o. and 200 mg/kg, p.o. of the FXM and its derived fractions; FXH, FXC, FXE, FXB and FXA. Latency period (time for which rat remains on the hot plate without licking or flicking of hind limb or jumping) in second was recorded for each animal of the group at 0 and after 30, 60 and 120 min of dose administration. In order to prevent the tissue damage the cut off time of 30 s was set for all animals. Percent analgesia was calculated using the following formula.

$$ \%\;\mathrm{Analgesia}=\left[\frac{\left(\mathrm{Test}\ \mathrm{latency}\hbox{--} \mathrm{control}\ \mathrm{latency}\right)}{\left(\mathrm{Cut}\ \mathrm{off}\ \mathrm{time}\hbox{--} \mathrm{control}\ \mathrm{latency}\right)}\times 100\right] $$

## Anti-inflammatory activity

### In vitro anti-inflammatory activities

#### Inhibition of TNF-α activated NFkB assay

Inhibition of TNF-α activated nuclear factor kappa-B (NFkB) assay was performed on 293/NFkB-Luc HEK cells purchased from Panomics (Freemont, CA, USA). Cells were seeded at a density of 2 × 10^4^ cells per 200 μl in sterile white-walled 96-well plate having Dulbecco’s Modified Eagle Medium supplemented with 10 % fetal bovine serum and antibiotic. After incubation for 48 h in 5 % CO_2_ at 37 °C, DMSO (10 %) was added without or with 0.5, 10, 15, 20, 25, 30, 40 μg/ml of the extract/fraction and 10 ng/ml of TNF-α at a final concentration in the fresh medium. The cells after incubation for 6 h at 37 °C were washed with PBS. Cells obtained were lysed in 50 μl of 1X reporter lysis buffer with one freeze/thaw cycle (−80 °C/37 °C). By using the Luciferase Assay System from Promega (Madison, WI, USA) the inhibition of NFkB was recorded in a luminometer according to the manufacturer’s instructions. In this assay cells not instigated with TNF-α were used as negative control whereas the cells not treated with DMSO and TNF-α were considered as positive control. The experiment was performed in three replications. After determination of percentage inhibition of NFkB the IC_50_ values were computed [44]. The data were then plotted graphically as dose response curve after changing the concentration of extract/fractions to the log scale.

### Inhibition of LPS-induced nitric oxide synthesis (nitrite assay)

To examine the anti-inflammatory effects of the crude methanol extract and fractions from leaves of *F. xanthoxyloides*, LPS-stimulated macrophage model was used [45]. RAW 264.7 cells (ATCC-TIB-71) were seeded at a density of 10 × 10^4^ cells per well in 96-well plate on 10 % FBS containing DMEM and incubated for 24 h at 37 °C. Extract/fractions in 10 % DMSO solution at a final concentration of 0.5, 10, 15, 20, 25, 30, 40 μg/ml in 1 % FBS-containing phenol red free DMEM were added and incubated further for 15 min at 37 °C. Cells were stimulated by the addition of 1 μg/ml of LPS and incubated further for 20 h at 37 °C. Cells not stimulated with LPS were considered as a negative control whereas cells treated with LPS and DMSO used as positive control. To determine the anti-inflammatory potential of extract/fractions, an aliquot of 100 μl of the incubation media was transferred to 96-well plate and allowed to react with Griess reagent (90 μl of 1 % sulfanilamide in 5 % phosphoric acid, and 90 μl of *N*-(1-naphthyl) ethylenediamine). Absorbance of the reaction mixture was recorded at 540 nm. The experiment was performed in three replications. After determination of percentage inhibition of nitric oxide IC_50_ values were computed. The data were then plotted graphically as dose response curve after changing the concentration of extract/fractions to the log scale.

### Cytotoxicity studies for dose response

Characterization of extract/fractions for cytotoxicity was demonstrated according to You et al. [46] by the sulforhodamine B assay. Briefly, 190 μl of cells with a density of 5 × 10^4^ cells/ml of both 293/NFkB-Luc HEK cells and RAW 264.7 cells (ATCC-TIB-71) were seeded in 96-well plate having 10 μl of the test sample (final concentration of 20 μg/ml) in DMSO (10 %) and PBS, and incubated for 72 h at 37 °C in CO_2_ incubator. After incubation 50 μl of 20 % TCA was added to terminate the reaction. Cells were washed, dried and stained with 0.4 % of acetic acid for 30 min at room temperature. Cells were washed four times with acetic acid and dried overnight. Bound dye was solubilized in 200 μl of 10 mM of Tris base (pH = 10) on a gyratory shaker for 10 min. Absorbance of each treatment was recorded at 515 nm through a micro-plate reader. A zero-day control was performed in each case following addition of equal quantity of cells in 16 wells, with subsequent incubation for 30 min at 37 °C and was processed as mentioned earlier. Cell survival percentage was calculated for each test sample. The data were presented graphically as dose response curve after changing the concentration of extract/fractions as log scale.

### Cytotoxicity studies for timed response

The dose response curve has indicated that FXC as compared to other extract/fractions had more effectively inhibited the synthesis of NFkB and NO in the respective assays. To establish the time response curve for cytotoxicity the FXC was used at concentration of 2× IC_50_, 1× IC_50_ and 1/2 × IC_50_ for 6, 12, 18, 24, 30 and 36 h. The experiment was performed in triplicate. The data obtained was graphically presented as time response curve.

### In vivo anti-inflammatory studies

#### Carrageenan induced paw edema

To assess the anti-inflammatory potential of the test samples carrageenan-induced inflammation model in rat was used [43]. Accordingly, Sprague-Dawley rats (150–200 g) were randomly assigned into 14 groups containing six rats each. At the primate facility of Quaid-i-Azam University Islamabad, rats were maintained at standard laboratory conditions and had free access to the laboratory feed and water *ad libitum*. Animals of various groups received 100 mg/kg and 200 mg/kg, p.o. of FXM and its derived fractions. For this study the standard drug used was diclofenac sodium at 10 mg/kg, p.o. whereas saline 2 ml, p.o. was given to negative control animals. After one hour of the extract/fractions or the standard drugs, rats were injected with 1 % of carrageenan into the plantar tissue of the right hind paw. Paw volume was measured plethysgraphically at 0^th^, 1^st^, 2^nd^, 3^rd^ and 4^th^ h after carrageenan injection and the following formulae for calculating percent inhibition of edema were used;

$$ \mathrm{E}\mathrm{V}=\mathrm{P}\mathrm{V}\mathrm{A}-\mathrm{P}\mathrm{V}\mathrm{I} $$

Where, EV = Edema volume, PVI = Paw volume before carrageenan administration (i.e. initial paw volume) and, PVA = Paw volume after carrageenan administration.

$$ \mathrm{Percent}\ \mathrm{inhibition}=\left[\frac{\left(\mathrm{E}\mathrm{V}\mathrm{c}\ \hbox{--} \mathrm{E}\mathrm{V}\mathrm{t}\right)}{\left(\mathrm{E}\mathrm{V}\mathrm{c}\right)}\times 100\right]. $$

EVc = Edema volume of control animals, EVt = Edema volume of test sample animals

### Subcutaneous air pouch

To determine the anti-inflammatory activity of FXC in air pouch model, Sprague-Dawley male rats (200–250 g) were divided into five groups having six rats in each. For the development of air pouch on back side of rat 20 ml of sterile air was injected on day 0, and re-injected 2 and 5 days later with 10 ml of sterile air. On the following day the rats orally received tested drugs (100 mg/kg p.o., 200 mg/kg p.o.), diclofenac sodium (10 mg/kg p.o.) as standard or vehicle (2.5 ml saline). Carrageenan solution (1 % in saline solution) of 1 ml was injected in the pouch 1 h after the administration of drugs. Animals were euthanized after 6 h and the pouch was washed with 3 ml of PBS.

### Cell count

To assess the leukocytes in the exudate, cell pellet was obtained by centrifugation at 500 × g (4 °C). Cell pellet was suspended in 1000 μl of PBS and after staining the leukocytes with hematoxylin and eosin (Sigma-Aldrich, MO, USA) the total cell number and differential cell count was made using a Neubauer hemocytometer. The supernatant was used for estimation of inflammatory mediators.

### Inflammatory mediators

The level of inflammatory mediators TNF-α and IL-6 was determined with commercial ELISA kits from OptEIA rat set while PGE_2_ was assessed by ELISA kit from Biotrend. The concentration was measured according to manufacturer’s instructions. The concentration of NO was estimated with Griess reagent (90 μl of 1 % sulfanilamide in 5 % phosphoric acid, and 90 μl of *N*-(1-naphthyl) ethylenediamine). Absorbance of the reaction mixture was recorded at 540 nm.

### Gas chromatography-mass spectrometry of FXM

GC-MS analysis was done by using GC-Mass spectrometer system (Model, Thermo GC-Trace ultra-version 5.0, Thermo MS DSQ-II, Thermo Fisher, USA), ZB 35-MS Capillary standard non-polar column (30 mts. X 0.25 mm, 0.25 m film thickness). Oven temperature programmed from 70 to 260 °C at 6 °C/min. The 1 μl/min volume of methanol extract sample was injected into the instrument and detected by splitless injection technique. The compounds were separated using Helium (1.0 ml/min) as the carrier gas [47]. The constituents present in the analyzed methanol leaf extract of *F. xanthoxyloides* were identified in comparison with their specters of mass with those gathered in a library search (NIST - MS) results and with those reported in the literature (Chemdata.nist.gov/).

### Statistical analysis

All the data obtained was reported as mean ± SD. One was analysis of variance was used to calculate the variation among various groups by using Statistix 8.1. Significant differences among groups were calculated by Tukey’s multiple comparison tests. Statistical significance was set at *P* >0.05. GraphPad Prism 5 was used to determine the IC_50_ values for in vitro studies.

## Results

### Peripheral analgesic effect

#### Acetic acid induced writhing test

A dose dependent response of the rats towards the count of writhes was exhibited by FXM and its fractions when subjected to the acetic acid induced writhing test (Table 1). FXC at 200 mg/kg, p.o. reduced the writhing count up to 77.23 ± 5.64 % as against the 80.04 ± 5.25 % and 73.00 ± 4.87 % of diclofenac sodium and aspirin, respectively. The inhibitory effect of diclofenac sodium and aspirin did not differ (*P* >0.05) from protective effect of FXC (200 mg/kg), in number of writhes induced with 1 % solution of acetic acid. The percent inhibition in writhes induced with other fractions at 200 mg/kg, p.o. exhibited following order; FXM > FXE > FXB > FXH > FXA.

**Table 1:** Effect of FXM and its fractions in acetic acid induced writhing test in rat

Groups	Drug (dose), route	No. of writhing (Mean ± SD)	% Inhibition
Saline	2 ml, i.p.	71.00 ± 2.52^a^	0
FXM	100 mg/kg, p.o.	30.50 ± 1.87^g^	57.04 ± 3.63
FXM	200 mg/kg, p.o.	22.00 ± 3.41^h^	69.01 ± 4.98
FXH	100 mg/kg, p.o.	63.50 ± 1.87^b^	10.56 ± 2.63
FXH	200 mg/kg, p.o.	51.00 ± 2.19^de^	28.16 ± 3.08
FXC	100 mg/kg, p.o.	39.16 ± 3.48^f^	44.83 ± 4.91
FXC	200 mg/kg, p.o.	16.16 ± 3.62^ij^	77.23 ± 5.64
FXE	100 mg/kg, p.o.	59.00 ± 1.41^bc^	16.90 ± 2.02
FXE	200 mg/kg, p.o.	48.00 ± 2.44^e^	32.39 ± 3.14
FXB	100 mg/kg, p.o.	55.66 ± 1.63^cd^	21.59 ± 2.30
FXB	200 mg/kg, p.o.	52.16 ± 1.16^de^	26.52 ± 1.64
FXA	100 mg/kg, p.o.	62.00 ± 1.78^b^	12.67 ± 2.51
FXA	200 mg/kg, p.o.	54.33 ± 1.75^cd^	23.47 ± 2.46
Diclofenac sodium	10 mg/kg, i.p.	14.16 ± 2.60^j^	80.04 ± 5.25
Aspirin	10 mg/kg, i.p.	19.16 ± 2.46^hi^	73.00 ± 4.87

*FXM F. xanthoxyloides* methanol extract, *FXH F. xanthoxyloides* n-hexanefraction, *FXC F. xanthoxyloides* chloroform fraction, *FXE F. xanthoxyloides* ethyl acetate fraction, *FXB F. xanthoxyloides* n-butanol fraction, *FXA F. xanthoxyloides* residual aqueous fraction. Data values shown represent mean ± SD (*n* = 6). One-way ANOVA followed by Tukey’s HSD multiple comparison tests. Different alphabets in each column indicate difference at *P* <0.05

### Central analgesic effect

#### Hot plate method (Thermal stimulation)

We performed the hot plate test, to assess the influence of *F. xanthoxyloides* crude extract (FXM) and its derived fractions on the thermal stimulation as central analgesic effect. Plant extract/fractions were used at 100 and 200 mg/kg dosages and latency time (seconds) was recorded at 0, 30, 60 and 120 min after the treatment of extract/fractions. Generally all the extract/fractions variably increased the latency time over dosages and time after the treatment (Table 2). Treatment of diclofenac sodium and morphine, 30 min after the drug treatment, induced >50 % analgesia by increasing the latency time of licking whilst; 60 min after the drug treatment, diclofenac sodium, morphine and FXM (200 mg/kg) exhibited >50 % analgesia. The response of the rats with FXM and FXC towards the thermal stimulation, 120 min after the administration of extract/fractions was significantly enhanced and maximum percent analgesia was exhibited by FXC (200 mg/kg) followed by FXM (200 mg/kg). Percent analgesia exhibited by FXC (200 mg/kg) was statistically similar (*P* >0.001) to the percent analgesia induced with diclofenac sodium and morphine, 120 min after the administration of the extract/fractions. Percent analgesia exhibited by other fractions (200 mg/kg), 120 min after the administration, was recorded in order of FXE > FXH > FXB > FXA.

**Table 2:** Effect of FXM and its fractions in hot plate test

Treatment	Dose/route	Mean latency time in seconds	Increase in latency time (seconds) after various dosages (Mean ± SD)/Percent analgesia		
		0 min	30 min	60 min	120 min
Saline	2 ml, i.p.	8.83 ± 0.75	10.33 ± 0.51	9.83 ± 0.40	9.50 ± 0.44
			7.03 ± 2.41^e^	4.65 ± 2.87^h^	3.03 ± 2.69^i^
FXM	100 mg/kg, p.o.	9.33 ± 0.51	13.00 ± 0.89	15.50 ± 0.54	17.00 ± 0.63
			17.61 ± 6.27^c^	29.80 ± 3.19^e^	37.10 ± 2.44^cd^
FXM	200 mg/kg, p.o.	9.66 ± 1.03	16.50 ± 0.54	21.33 ± 1.03	23.66 ± 1.03
			33.46 ± 4.41^b^	57.35 ± 4.72^c^	68.72 ± 5.71^b^
FXH	100 mg/kg, p.o.	9.50 ± 0.54	10.33 ± 0.51	12.00 ± 0.89	13.66 ± 0.51
			4.00 ± 3.59^de^	12.10 ± 5.78^fgh^	20.31 ± 1.77^fgh^
FXH	200 mg/kg, p.o.	9.50 ± 0.54	10.66 ± 0.51	13.66 ± 0.51	15.66 ± 0.51
			5.63 ± 3.56^de^	20.31 ± 1.77^ef^	30.00 ± 4.08^def^
FXC	100 mg/kg, p.o.	9.16 ± 0.75	12.33 ± 0.51	15.00 ± 0.89	18.00 ± 0.89
			15.13 ± 3.23^cd^	27.99 ± 3.46^e^	42.33 ± 4.83^c^
FXC	200 mg/kg, p.o.	9.00 ± 0.63	15.66 ± 0.51	18.66 ± 0.51	25.00 ± 0.89
			31.69 ± 3.14^b^	45.99 ± 3.89^d^	76.13 ± 4.49^ab^
FXE	100 mg/kg, p.o.	9.66 ± 0.51	12.00 ± 0.89	12.33 ± 0.51	13.33 ± 0.51
			11.46 ± 4.01^cde^	13.05 ± 3.72^fgh^	18.01 ± 2.37^fgh^
FXE	200 mg/kg, p.o.	9.66 ± 0.81	12.33 ± 0.51	14.66 ± 0.51	16.66 ± 0.89
			13.02 ± 3.81^cd^	24.54 ± 2.44^e^	34.39 ± 3.24^cde^
FXB	100 mg/kg, p.o.	9.33 ± 1.03	10.33 ± 0.72	12.33 ± 0.51	14.00 ± 0.89
			4.60 ± 2.11d^e^	14.42 ± 2.43^fg^	22.53 ± 3.36^fgh^
FXB	200 mg/kg, p.o.	9.83 ± 0.75	11.16 ± 0.89	12.83 ± 0.51	15.00 ± 0.89
			5.63 ± 3.42^de^	10.59 ± 6.40^gh^	25.53 ± 5.14^efg^
FXA	100 mg/kg, p.o.	9.50 ± 0.54	9.66 ± 0.51	11.66 ± 0.51	12.33 ± 0.51
			3.75 ± 0.65^c^	10.47 ± 4.55^gh^	13.77 ± 3.41^h^
FXA	200 mg/kg, p.o.	9.00 ± 0.89	10.33 ± 0.51	12.00 ± 0.89	13.66 ± 1.03
			6.13 ± 4.21^cde^	14.30 ± 1.61^fg^	22.25 ± 2.68^fgh^
Diclofenac sodium	10 mg/kg, i.p.	9.83 ± 0.75	21.66 ± 0.51	26.66 ± 1.51	26.66 ± 1.62
			58.69 ± 1.45^a^	83.51 ± 4.21^a^	83.39 ± 5.07^a^
Morphine	10 mg/kg, i.p.	9.33 ± 0.51	20.16 ± 0.75	23.66 ± 0.81	23.00 ± 0.89
			52.34 ± 4.59^a^	69.36 ± 3.84^b^	66.03 ± 5.13^b^

*FXM F. xanthoxyloides* methanol extract, *FXH F. xanthoxyloides* n-hexanefraction, *FXC F. xanthoxyloides* chloroform fraction, *FXE F. xanthoxyloides* ethyl acetate fraction, *FXB F. xanthoxyloides* n-butanol fraction, *FXA F. xanthoxyloides* residual aqueous fraction. Data values shown represent mean ± SD (*n* = 6). One-way ANOVA followed by Tukey’s HSD multiple comparison tests. Percentage analgesia is shown in brackets. Different alphabets in each column indicate difference at *P* <0.05

### In vitro anti-inflammatory activities

#### Inhibition of TNF-α activated nuclear factor kappa-B (NFkB assay)

**Fig. 1:**
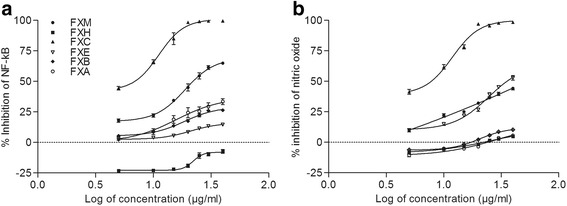
Dose response curve of NF-kB (**a**) and nitric oxide (**b**) synthesis for various extract/fractions of F. xanthoxyloides. *FXM Fraxinus xanthoxyloides* methanol extract of leaves, *FXH Fraxinus xanthoxyloides* n-hexane fraction of methanol extract of leaves, *FXC Fraxinus xanthoxyloides* chloroform fraction of methanol extract of leaves, *FXE Fraxinus xanthoxyloides* ethyl acetate fraction of methanol extract of leaves, *FXB Fraxinus xanthoxyloides* n-butanol fraction of methanol extract of leaves, *FXA Fraxinus xanthoxyloides* residual aqueous fraction of methanol extract of leaves. The values above ‘0’ point intersect indicate inhibition while below represent stimulation

**Fig. 2:**
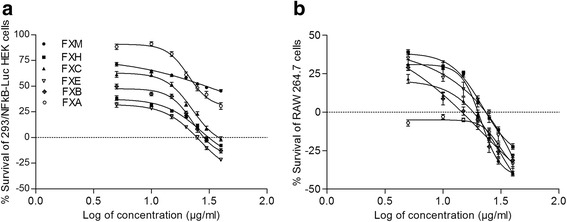
Dose response curve of % survival of 293/NFkB-Luc HEK cells (**a**) and RAW 264.7 cells (**b**) for various extract/fractions of *F. xanthoxyloides. FXM Fraxinus xanthoxyloides* methanol extract of leaves, *FXH Fraxinus xanthoxyloides* n-hexane fraction of methanol extract of leaves, *FXC Fraxinus xanthoxyloides* chloroform fraction of methanol extract of leaves, *FXE Fraxinus xanthoxyloides* ethyl acetate fraction of methanol extract of leaves, *FXB Fraxinus xanthoxyloides* n-butanol fraction of methanol extract of leaves, *FXA Fraxinus xanthoxyloides* residual aqueous fraction of methanol extract of leaves. The values above ‘0’ point intersect indicate stimulation while below represent inhibition

The present study demonstrate the inhibitory potential of the crude methanol extract and its fractions of the leaves of *F. xanthoxyloides* on TNF-α instigated NFkB on 293/NFkB-Luc HEK cells (Fig. 1a). The extract/fractions variably inhibited the NFkB except the FXH where up-regulation of NFkB activity was recorded at all concentrations. The most potent inhibitor of NFkB activity was the FXC and it has effectively inhibited the activity (85.0 ± 8.12 %) at 15 μg/ml concentration. The inhibitory effect at the same dose followed by other extract/fractions was FXM (34.46 ± 1.86 %) > FXA (18.8 ± 5.61 %) > FXB (13.7 ± 0.86 %) > FXE (5.6 ± 0.70 %). FXC had demonstrated IC_50_ value of 5.98 μg/ml. Survival percentage recorded by sulforhodamine B assay indicated that FXA only induced the cytotoxicity at all the doses. All the other extract/fractions used in this experiment did not induce cytotoxicity up till 20 μg/ml concentration in the medium (Fig. 2a).

### Inhibition of LPS-induced NO production (nitrite assay)

In this experiment nitric oxide (NO) production was induced with the application of LPS during in vitro conditions and estimation of nitrite; the major oxidized metabolite of nitric oxide was recorded (Fig. 1b). Application of FXC was the most effective and inhibited the production of NO at 15 μg/ml (78.23 ± 2.91 %) with IC_50_ value of 6.59 μg/ml. Percentage inhibition in NO production recorded for FXM and FXE at 15 μg/ml was 25.06 ± 1.35 %, 21.96 ± 2.71 % respectively. On the other hand FXH, FXE and FXA did not inhibit the synthesis of nitric oxide at the same concentration. As far as the cytotoxicity is concerned FXA induce cytotoxicity at all the concentrations used in this experiment. FXH, FXC and FXE at lower doses (up till 15 μg/ml) did not cause death of the cells (Fig. 2b).

### Time dose response studies

**Fig. 3:**
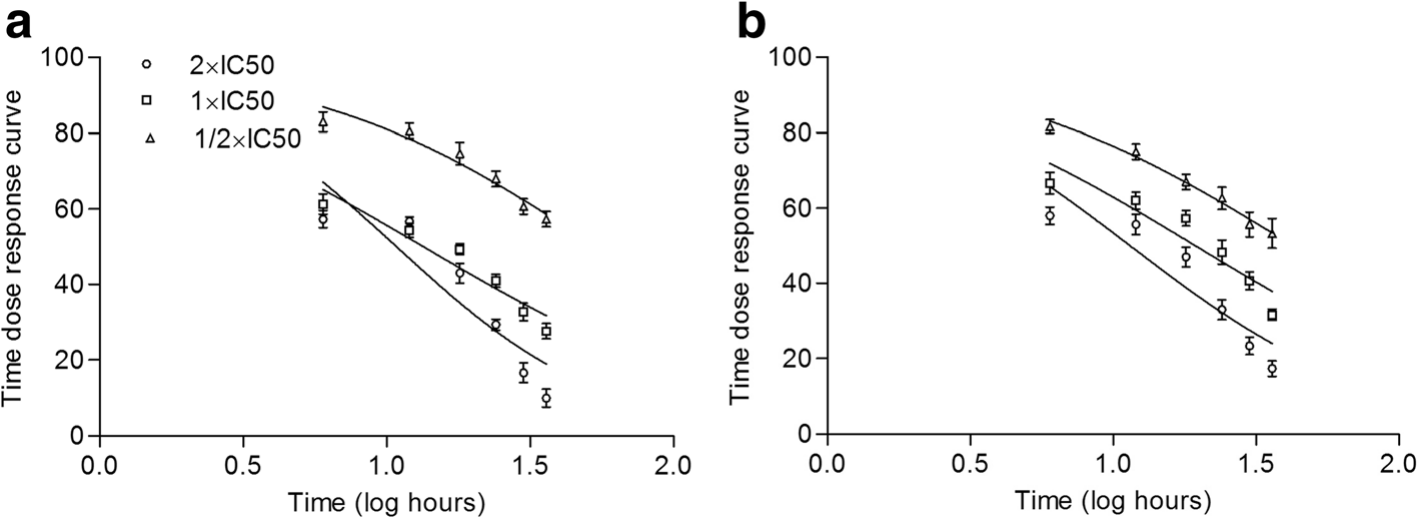
Time dose response curve of % survival 293/NFkB-Luc HEK cells (**a**) and RAW 264.7 cells (**b**) for various concentration of FXC. All the concentrations exhibited stimulation. ‘0’ point intersect indicates 100% survival

The FXC was able to effectively inhibit the activity of NFkB and nitric oxide synthesis in the respective assays. On the basis of IC_50_ obtained for both assays three concentrations; 2 × IC_50_, 1 × IC_50_, 1/2 × IC_50_ were used to estimate the cytotoxicity at various intervals of exposure. The data obtained for time-dose response indicated that these doses did not induce cytotoxicity rather stimulated the respective cells at all the times of treatment. However all the concentrations reduced the stimulation of cell proliferation with the increase of time (Fig. 3).

### Carrageenan induced paw edema

To demonstrate the anti-inflammatory activity of the extract/fractions of *F. xanthoxyloides* leaves we have used the carrageenan for induction of edema in hind paw of rat. Rats were treated with carrageenan and various extract/fractions, and the anti-inflammatory response was assessed by measuring the volume of hind paw over concentration and time (Table 3). Crude extract of *F. xanthoxyloides* (FXM) and its fractions (FXH, FXC, FXE, FXB, FXA), induced a dose and time dependent reduction in paw edema. Percent anti-inflammatory effects produced after I h of the injection of carrageenan, by FXC (200 mg/kg) and FXM (200 mg/kg) were significantly (*P* <0.001) higher as compared to the diclofenac sodium, standard drug used in this study. However, 2, 3 and h after the carrageenan induced paw edema, FXC (200 mg/kg) exhibited statistically similar (*P* >0.001) percent inhibition of edema to that of the diclofenac sodium. The following order of edema inhibition FXM > FXE > FXB > FXH > FXA was recorded for rest of the fractions at the maximum dose (200 mg/kg) after 4 h of the carrageenan treatment.

**Table 3:** Effect of FXM and its fractions on carrageenan induced paw edema in rat

Treatment	Dose/route	Mean paw volume before carrageenan injection	Increase in paw volume (ml) after carrageenan injection (Mean ± SD)/Percent inhibition of edema			
			+1 h	+2 h	+3 h	+4 h
Saline	2 ml, i.p.	1.12 ± 0.08	2.58 ± 0.09	2.67 ± 0.09	2.21 ± 0.09	1.81 ± 0.11
FXM	100 mg/kg, p.o.	1.11 ± 0.08	2.52 ± 0.06	2.30 ± 0.09	2.12 ± 0.20	1.48 ± 0.08
			5.88 ± 1.99^c^	19.72 ± 2.39^cde^	35.84 ± 12.89^cd^	45.28 ± 4.02^bc^
FXM	200 mg/kg, p.o.	1.17 ± 0.09	2.44 ± 0.04	2.05 ± 0.06	1.92 ± 0.04	1.45 ± 0.06
			15.34 ± 7.95^b^	41.07 ± 5.00^b^	52.51 ± 8.30^b^	58.52 ± 8.19^b^
FXH	100 mg/kg, p.o.	1.15 ± 0.08	2.61 ± 0.08	2.56 ± 0.09	2.51 ± 0.11	1.71 ± 0.08
			2.84 ± 2.42^c^	4.99 ± 3.22^g^	13.44 ± 5.41^e^	18.81 ± 2.21^e^
FXH	200 mg/kg, p.o.	1.11 ± 0.05	2.55 ± 0.05	2.33 ± 0.06	2.32 ± 0.05	1.61 ± 0.04
			3.96 ± 2.28^c^	17.89 ± 2.03^de^	23.29 ± 4.53^de^	27.88 ± 6.75^de^
FXC	100 mg/kg, p.o.	1.14 ± 0.06	2.58 ± 0.06	2.21 ± 0.06	2.06 ± 0.13	1.53 ± 0.08
			3.85 ± 1.85^c^	28.28 ± 3.11^c^	42.12 ± 7.49^bc^	43.57 ± 5.21^bc^
FXC	200 mg/kg, p.o.	1.09 ± 0.04	2.17 ± 0.04	1.71 ± 0.03	1.53 ± 0.04	1.25 ± 0.04
			29.19 ± 4.60^a^	59.67 ± 3.23^a^	73.29 ± 2.65^a^	77.64 ± 3.04^a^
FXE	100 mg/kg, p.o.	1.12 ± 0.07	2.59 ± 0.06	2.47 ± 0.04	2.34 ± 0.06	1.55 ± 0.08
			1.71 ± 0.82^c^	8.76 ± 3.66^fg^	22.32 ± 6.02^de^	36.22 ± 8.23^cd^
FXE	200 mg/kg, p.o.	1.12 ± 0.06	2.56 ± 0.07	2.25 ± 0.05	2.18 ± 0.05	1.49 ± 0.05
			4.08 ± 1.78^c^	23.60 ± 2.10^cd^	32.38 ± 4.66^cd^	45.77 ± 4.22^bc^
FXB	100 mg/kg, p.o.	1.11 ± 0.06	2.59 ± 0.04	2.50 ± 0.05	2.32 ± 0.07	1.62 ± 0.07
			1.49 ± 1.71^c^	6.25 ± 1.15^fg^	23.07 ± 4.45^de^	25.68 ± 3.08^de^
FXB	200 mg/kg, p.o.	1.11 ± 0.06	2.55 ± 0.07	2.30 ± 0.06	2.19 ± 0.08	1.52 ± 0.06
			3.63 ± 1.31^c^	19.84 ± 3.34^cde^	31.08 ± 5.33^cd^	39.89 ± 7.36^cd^
FXA	100 mg/kg, p.o.	1.10 ± 0.06	2.58 ± 0.06	2.48 ± 0.09	2.50 ± 0.08	1.70 ± 0.05
			1.60 ± 1.16^c^	7.05 ± 2.71^fg^	11.17 ± 2.82^e^	13.18 ± 3.04^e^
FXA	200 mg/kg, p.o.	1.12 ± 0.06	2.59 ± 0.05	2.39 ± 0.04	2.36 ± 0.07	1.63 ± 0.01
			1.82 ± 1.64^c^	14.47 ± 4.31^ef^	20.80 ± 2.55^de^	24.94 ± 7.76^de^
Diclofenac sodium	10 mg/kg, i.p.	1.13 ± 0.08	2.57 ± 0.05	1.63 ± 0.05	1.46 ± 0.08	1.24 ± 0.04
			4.08 ± 2.47^c^	67.32 ± 4.25^a^	80.54 ± 2.61^a^	85.23 ± 7.71^a^

*FXM F. xanthoxyloides* methanol extract, *FXH F. xanthoxyloides* n-hexanefraction, *FXC F. xanthoxyloides* chloroform fraction, *FXE F. xanthoxyloides* ethyl acetate fraction, *FXB F. xanthoxyloides* n-butanol fraction, *FXA F. xanthoxyloides* residual aqueous fraction. Data values shown represent mean ± SD (*n* = 6). One-way ANOVA followed by Tukey’s HSD multiple comparison tests. Percentage inhibition is shown in brackets. Different superscript alphabets in each column indicate difference at *P* <0.05

### Subcutaneous air pouch assay

#### Effect of FXC on the hematology of air pouch exudate

In this study both doses of FXC significantly (*P* <0.001) decreased the count of neutrophils, monocytes, lymphocytes and WBCs in the exudate of air pouch induced with carrageenan (Table 4). Decrease in the number of neutrophils and monocytes in the exudate treated with FXC at 200 mg/kg and diclofenac sodium was statistically (*P* >0.001) similar to each other. However, number of lymphocytes and WBCs in the exudate was more markedly (*P* <0.001) decreased to that of the diclofenac sodium treated rats.

**Table 4:** Effect of FXC on the hematology (×10^3^/μl) in carrageenan induced exudate in the air pouch

	Dose/route	Neutrophils	Monocytes	Lymphocytes	WBCs
Saline	2 ml i.p.	0.150 ± 0.009^d^	0.079 ± 0.010^d^	0.472 ± 0.040^d^	0.945 ± 0.0982^d^
Carrageenan	10 ml/kg p.o.	6.027 ± 0.350^a^	1.972 ± 0.136^a^	12.665 ± 1.456^a^	23.422 ± 1.556^a^
Diclofenac sodium	10 mg/kg p.o.	4.494 ± 0.172^c^	1.445 ± 0.114^bc^	10.978 ± 1.231^ab^	17.521 ± 1.201^b^
FXC	100 mg/kg p.o.	5.082 ± 0.238^b^	1.665 ± 0.125^b^	9.240 ± 0.857^b^	16.932 ± 0.974^b^
FXC	200 mg/kg p.o.	4.285 ± 0.153^c^	1.353 ± 0.122^c^	5.698 ± 0.483^c^	12.199 ± 1.571^c^

FXC = *F. xanthoxyloides* chloroform fraction. Count of cells is presented as mean ± SD (*n* = 6). One way analysis of variance for count of cells was followed by multiple comparisons by Tukeys’ HSD test. Superscript alphabets indicate significance among treatments (*P* <0.001) on not sharing common letter

### Effect of FXC on inflammatory mediators of air pouch exudate

The results obtained for the alteration in inflammatory mediators of air pouch exudate are presented in Table 5. The results indicated a dose dependent decrease in the inflammatory mediators; TNF-α, IL-6, NO and PGE_2_ with FXC in the exudate of air pouch of rat induced with carrageenan injection. In this experiment generally a dose dependent decrease in the level of inflammatory mediators with FXC was recorded in the air pouch exudate of rat. Level of TNF-α and NO in the air pouch exudate was found higher at 200 mg/kg of FXC treatment but was statistically (*P* >0.001) similar to that of the diclofenac sodium treatment. On the other hand the level of IL-6 and PGE_2_ was recorded at lower level but statistically (*P* >0.001) similar as compared to the diclofenac sodium treatment.

**Table 5:** Inhibitory effect of FXC on inflammatory mediators in exudate of air pouch

			Estimated values/(Percent inhibition)		
	Dose/route	TNF-α (pg/ml)	IL-6 (pg/ml)	NO (μM/ml)	PGE_2_ (pmole/ml)
Saline	2 ml i.p.	5.50 ± 1.04^d^	27.66 ± 5.00^c^	0.443 ± 0.058^d^	1301.8 ± 90.87^b^
Carrageenan	10 ml/kg p.o.	964.50 ± 95.525^a^	6291.3 ± 562.77^a^	0.784 ± 0.105^a^	1525.7 ± 75.76^a^
Diclofenac sodium	10 mg/kg p.o.	418.50 ± 57.83^c^	4544.8 ± 235.87^b^	0.362 ± 0.043^c^	1339.7 ± 25.17^b^
		56.44 ± 6.01	34.03 ± 3.42	56.11 ± 4.99	7.69 ± 1.73
FXC	100 mg/kg p.o.	592.33 ± 45.31^b^	5146.2 ± 257.58^b^	0.605 ± 0.060^b^	1404.7 ± 21.37^ab^
		38.35 ± 4.71	25.30 ± 3.73	26.71 ± 7.33	3.21 ± 1.47
FXC	200 mg/kg p.o.	505.67 ± 36.037^bc^	4398.2 ± 159.46^b^	0.408 ± 0.049^bc^	1309.8 ± 30.46^b^
		47.37 ± 3.75	36.16 ± 2.31	50.52 ± 5.96	9.74 ± 2.09

FXC *F. xanthoxyloides* chloroform fraction, tumor necrosis factor- α (TNF-α), interleukin-6 (IL-6), nitric oxide (NO), prostaglandin E_2_ (PGE_2_). Count of cells is presented as mean ± SD (*n* = 6). One way analysis of variance for inflammatory mediators was followed by multiple comparisons by Tukeys’ HSD test. Superscript alphabets indicate significance among treatments (*P* <0.001) on not sharing common letter

### Gas chromatography-mass spectrometry of FXM

In the crude methanol extract of *F. xanthoxyloides*, 30 compounds were detected through GC-MS analysis (Table 6). The chromatogram showed prominent peaks in the retention time range 3.80–38.54 (Fig. 1). The GC-MS analysis provided six major peaks determining the presence of 15 major classes of compounds contributing three terpenoids (26.61 %), four lactam (16.47 %), three esters (15.81 %), three phenols (8.37 %), two steroid (6.91 %), three alcohols (5.02 %), three ketones (4.49 %), one aldehyde (3.89 %), two fatty acid glycerol (3.01 %), one nitrile (2.64 %), two lactones (2.31 %), one silyl-ether (2.25 %), one alkene (1.31 %) and one alkyne (0.89 %).

**Table 6:** GC-MS analysis of FXM

	Compound name	Area %	Class
1.	7-(diphenylmethylene) -6-phenylbicyclo [3.2.0] hept-2-en-endo-6-ol	2.32	Alcohol
2.	1-[1-(Bromomethyl) 2 (2,2,2-trichloroethyl) cyclopent-5-y-l]-(1H,3H)-5-methyl-pyrimidine-2,4-dione	1.15	δ Lactam
3.	2,2-Dimethyl-5-(2′,2′-dideuterio-1′-indanyldene)-1,3-dioxane-4,6-dion	1.00	Lactone
4.	3-Heptene, (E)- (CAS)	1.31	Alkene
5.	(S)-(−)-2-(Methoxymethoxy) propan-1-al	3.89	Aldehyde
6.	(S)-5-(1-Hydroxy-1-methylethyl)-2-pyrrolidinone	4.13	γ Lactam
7.	1-(3,6,6-Trimethyl-1,6,7,7a-tetrahydrocyclopenta [c] pyran-1-yl)ethanone	2.10	Ketone
8.	Phenol, 4-nitroso- (CAS)	1.78	Phenol
9.	1-(0-Methoxyphenyl)-3-Buten-1-ol	1.04	Alcohol
10.	5-Isopropyl-4 (trifluoromethyl)-1H-pyrimidin-2-one	7.28	δ Lactam
11.	9-Hydroxy-1-methyl-1,2,3,4-tetrahydro-8 h-pyrido (1,2-A) pyrazin-8-one	0.85	Ketone
12.	á-ionol	1.66	Alcohol
13.	Methyl (E)-5-(2 Oxocyclopentyl)-2-pentenoate ethylene ketal	2.61	Ester
14.	4-((1E)-3-Hydroxy-1-propenyl)-2-methoxyphenol	5.44	Phenol
15.	(E)-3-(3′-hydroxy-2′,6′,6′-trimethyl-1′-cyclohexen-1′-yl propionic acid methyl ester	11.98	Ester
16.	Isoquinolinium bis (ethoxycarbonyl) methylide	1.22	Ester
17.	1-Cyclododecanone, 2-ethylidene	1.54	Ketone
18.	3,5-Dimethoxy-p-coumaric alcohol	1.15	Phenol
19.	2-Hexadecen-1-ol,3,7,11,15-tetramethyl-,[R-[R*,R*-(E)]] (CAS) or (Phytol)	4.92	Diterpene
20.	2,4,6-Trimethoxyisophthalonitrile	2.64	Nitrile
21.	1-Phenyl-2-(3′,4′,5′ trimethoxyphenyl) ethyne	0.89	Alkyne
22.	2H-Pyran-2-one, tetrahydro-6-tridecyl- (CAS)	1.31	Lactone
23.	Pyrimidine, 5-methyl-2,4-bis[(trimethylsilyl)oxy]- (CAS)	2.25	Silylether
24.	2-palmitoyl glycerol	1.32	Fatty acid glycerol
25.	Gusation A	3.91	Lactam
26.	2-linoleoyl glycerol	1.69	Fatty acid glycerol
27.	Cholest-5-ene-1,3,16-triol, (1à,3á,16á)- (CAS)	1.08	Steroid
28.	Squalene	18.95	Triterpene
29.	Stigmasta-5,22-dien-3-ol, (3á,22E)- (CAS)	5.83	Steroid
30.	6,10,14,18,22-Tetracosapentaen-2-ol 3-bromo 2,6,10,15,19,23-hexamethyl-, (all-E)-	2.74	Terpenoid

## Discussion

Nowadays it is an accepted view that an unbalanced diet, environmental stresses and related anomalies increased the inclination of the affected individuals towards the chronic ailments such as cardiovascular, arthritis and diabetes [48]. Inflammation, pain and associated symptoms are all attributed to the elevated level of prostaglandins, tumor necrosis factor and interleukins [2]. To evaluate the peripheral analgesia of drugs or herbal products, acetic acid induced pain model is mostly used as it involves the peripheral nociceptors. Enhanced level of free arachidonic acid, prostaglandins especially PGE_2_ and PGE_2_α, and lipoxygenase in the peritoneal fluid, produce pain sensation as an inflammatory effect of acetic acid. Writhing paradigm is elicited due to stimulation of pain at nerve endings on account of swelling and vascular permeability. Activity of COX is decreased due to NSAIDs such as diclofenac sodium in the peripheral tissues results in inhibition of transduction of key afferent nociceptors [49]. In our study count of writhes was decreased in a dose dependent fashion and was dominantly decreased at 200 mg/kg, p.o. of FXC which was comparable to the standard drugs diclofenac sodium and aspirin (Table 1). From these results it can be hypothesized that FXC might possess some pharmacologically active metabolites that interfere with the synthesis of prostaglandins. However, it is worthwhile to investigate the exact metabolic event where the extract exerts its anti-nociceptive effects.

Treatment of rats with FXC at 200 mg/kg, p.o. in hot plate thermal stimulation model, exhibited beneficial analgesic effects at 120 min after treatment. In general the analgesic effect recorded was dose dependent and the latency time was enhanced by increase of time for all the test samples (Table 2). Thermal nociception model such as hot plate provide valuable clues about the analgesic activity of the test samples. The analgesic effect recorded in this study might involve the same mechanism of action; activation of opioid receptors, by the test samples similar to the diclofenac sodium. On account of apparent similarity of the analgesic effect of the test samples and the diclofenac sodium, implicate that test samples behave as narcotic analgesic and might work in the same manner to reduce pain sensation as diclofenac sodium [49]. In this study the diclofenac sodium and morphine (10 mg/kg, i.p.) exhibited the analgesic threshold after 30 min and more or less attained the maximum level after 60 min of treatment. However, FXC (200 mg/kg, p.o.) attained the maximum analgesic threshold after 120 min of treatment. Secondly, FXC (200 mg/kg, p.o.) exhibited higher analgesic activity to that of morphine, 120 min after the treatment. These results implicated a difference of action between the morphine and the test samples, as the herbal extracts constitute varied combination of metabolites. The active metabolites of the FXC might interfere with the release of pain mediators.

NFkB is an inducible transcription factor that regulates the fate of cells such as programmed cell death, proliferation control, cell invasion and in tumorigenesis. Over expression of NFkB through TNF-α during in vitro conditions provides an opportunity to unravel the potential of natural products to inhibit the immune response provoked by a variety of stresses. In this study, 293/NFkB-Luc HEK cells were stimulated by TNF-α for the synthesis of NFkB in the presence of FXM and its fractions; FXH, FXC, FXE, FXB and FXA at 0.5,10, 15, 20, 25, 30, 40 μg/ml concentration (Fig. 1a). Among the extract/fractions, FXC has shown the highest inhibitory activity at 15 μg/ml 85.00 ± 8.12 % (IC_50_ = 5.98 μg/ml) against NFkB followed by FXM (34.46 ± 1.22 %, IC_50_ = 23.01 μg/ml) whereas FXH (−21.80 ± 1.22 %) enhanced the synthesis of NFkB. It is conceivable that FXC might inhibit certain stimuli such as oxidative damage or inflammatory cytokines as a mechanism to inhibit NFkB signaling. Strong anti-NFkB activity has also been determined in chloroform fraction of *Carpinus tschonoskii* leaves [50]. Further, in this study cytotoxicity was not induced with the extract/fractions of *F. xanthoxyloides* at the same dose level. In addition, inhibition of NFkB signaling is considered to be an effective step in anticancer properties because up-regulation of NFkB suppresses apoptosis [51]. The results obtained in this study suggest the therapeutic importance of FXC and FXM as a natural remedy for various inflammatory disorders [52].

Early stages of inflammation predominantly results in the up-regulation of inducible enzymes such as COX-2 and iNOS which subsequently enhance the release of pro-inflammatory mediators such as TNF-α and NO [1]. Nitric oxide is one of the factors involved in the propagation of inflammatory responses. It also mediates a variety of functions involving vascular homeostasis, neurotransmission and host defense [1]. Nitric oxide is highly reactive molecule that act as an important mediator and regulator of inflammatory responses. Nitric oxide has the potential to react with superoxide anion to produce the highly reactive, free radical peroxynitrite which can cause irreversible damage to cell membranes, leading to cell death and tissue damage [53]. Nitric oxide induced oxidative stress is one the causal agent towards many chronic ailments such as diabetes, vascular disorders, and Parkinson’s disease [54]. In the presence of such disorders altering the macrophage nitric oxide synthesis could potentially attenuates a variety of inflammatory mediators. LPS-stimulated macrophage model was used in this study to investigate the efficacy of the tested samples. The results exhibited that FXC at 15 μg/ml was highly effective in inhibiting the generation of nitric oxide (78.23 ± 2.90 %, IC_50_ = 6.59 μg/ml) whereas no considerable inhibitory activity at the same dose was detected in crude extract and other fractions (Fig. 1b). Inhibition of nitric oxide production in LPS-induced nitric oxide model with chloroform fraction of *Cudrania tricuspidata* [55] and with extract of *Salvia officinalis* in RAW 264.7 macrophages have been reported earlier [1].

Carrageenan, phlogistic agent, is widely used to induce paw edema in rodents to demonstrate anti-inflammatory effect of drugs or herbs. Carrageenan when injected in the rat paw it induces severe edema that is discernible within 30 min [56]. In the present investigation test samples at 100 and 200 mg/kg, p.o. were subjected for their anti-inflammatory activity in a carrageenan-induced paw edema model in rat. FXC at dose level of 200 mg/kg, p.o. efficiently inhibited the edema formation at 1st, 2nd, 3rd and 4th h after the treatment. The anti-inflammatory activity of FXC was recorded significantly (*P* <0.001) higher to the drugs diclofenac sodium, at 1 h after the treatment. The lower dose (100 mg/kg, p.o.) of FXM and FXC while, higher dose (200 mg/kg, p.o.) of FXE and FXB exerted moderate anti-inflammatory activity (Table 3). Carrageenan-induced inflammation is considered to be a biphasic model. Initial stage (1–2 h) contributes to the release of histamine, bradykinin and serotonin which mediates the increased synthesis of prostaglandins from surrounding tissues of the injured area [49]. During second phase (3–4 h) inflammation is sustained by the perpetual release of prostaglandins [57]. In this phase COX-2 enzyme plays a key role to sustain inflammation by converting arachidonic acid into prostaglandins. These substances are responsible for the formation of inflammatory exudates. Most of the NSAIDs, such as diclofenac sodium do not inhibit the formation of pro-inflammatory mediators and substances of the primary phase whereas target the COX-2 enzyme and inhibit the paw edema during later stage of inflammatory response [57]. In this study FXM and FXC inhibited the paw edema in both phases (0–4 h), suggesting the stabilization of the lysosomal membrane during initial phase which in turn inhibit the release of inflammatory mediators. The efficient inhibition of inflammation by FXC, FXM and moderate inhibition of inflammation by FXE and FXB at 200 mg/kg dose has been determined during late phase after carrageenan treatment. It can be speculated that active leads present in the extract/fraction might inhibit the activity of COX enzyme thereby attenuates the conversion of arachidonic acid to prostaglandins. Although the mechanism of edema inhibition of *F. xanthoxyloides* during early phase in not known, it might be attributed by the inhibition of NO production and NFkB signaling as we have determined during in vitro studies. On the basis of these observations we may assume that diverse active phyto-constituents present in FXC show non-selective interaction, greater half-life and synergistic effects towards the inhibition of inflammatory response.

On account of significant anti-inflammatory activity during in vitro and in vivo experiments, FXC was used in the air pouch inflammatory model to assess its anti-inflammatory potential on the inflammatory mediators and the leukocyte count. Development of air pouch in rat resembles the inflamed synovial membrane of patients with rheumatoid arthritis. In this experiment the increase in the count of neutrophils, monocytes, leukocytes and the WBCs in the air pouch exudate with carrageenan was substantially decreased (*P* <0.001) with both the doses of FXC (Table 4). The migration of polymorphonuclear leukocytes in the pouch was inhibited by FXC and the effect produced by the higher dose of FXC (200 mg/kg) was similar to the diclofenac sodium. The migration of leukocytes towards the injured area is coupled with the release of inflammatory mediators such as NO, IL-6 and TNF-α [58]. In the air pouch exudate substantial increase in the level of these inflammatory mediators has been recorded indicating the development of inflammation at the injured site in rat (Table 5). However, the marked decrease in carrageenan induced accumulation of NO, IL-6 and TNF-α by FXC in the air pouch exudate reflected the repairing potential of the FXC. In general the enhanced level of NO at the inflamed site is usually achieved by activation of the inducible nitrate synthase (iNOS) activity. The results obtained in this study suggested that the blockage of TNF-α-NO pathway by FXC might occur through the inhibition of inducible nitrate synthase (iNOS) activity.

Prostaglandins at the injured area are produced as an effect of COX-2 from arachidonic acid and are considered to be involved in the endurance of inflammation, pain and pyrexia [59]. In the air pouch exudate the level of PGE_2_ was markedly elevated in the carrageenan treated rats. However, the use of FXC to carrageenan treated rats markedly inhibited the release of PGE_2_ in the air pouch exudate. These results suggest that the decreased level of PGE_2_ with FXC in the carrageenan induced air pouch exudate can either be achieved through the inhibition of COX-2 enzyme and/or the suppression of inflammation related cellular activities.

Our results of acute toxicity studies suggest that the crude extract of the leaves of *F. xanthoxyloides* and its fractions was safe in and non-toxic to rats at 3000 mg/kg dosages. So the use of 100 and 200 mg/kg of the extract/fractions was selected for various in vivo studies.

**Fig. 4:**
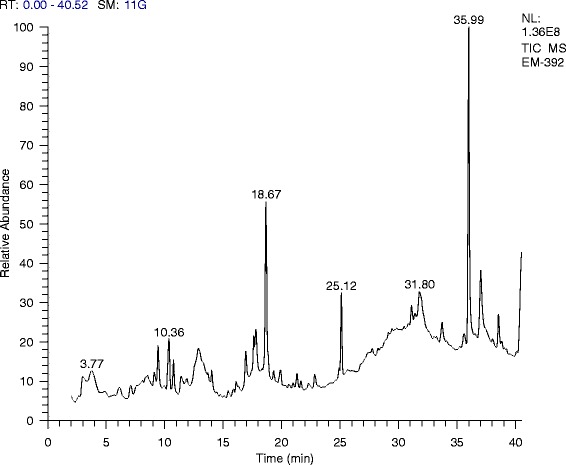
GC-MS analysis of the methanol extract of *F. xanthoxyloides*

Phytochemical profile obtained through GC-MS analysis indicated that FXM is comprised of varied compounds belonging to 15 major classes (Table 6). The retention times obtained for various constituents are presented in Fig. 4. The main class was comprised of terpenoids, and the rest of the classes include the lactams, esters, phenols, steroids, alcohols, and ketones. The anti-inflammatory activity of the extract/fractions might be attributed due the presence of sterols and terpenoids [60]. The difference in activity of the extract/fractions obtained in this study might involve the varied combination of compounds and their concentration in the respective extract or fraction. Among the phyto-constituents reported through GC-MS analysis; squalene is natural triterpenoid that targets pro-inflammatory mediators, modulate NFkB signaling and prevent over activation of macrophages, neutrophils and monocytes. It has exhibited anti-inflammatory activities in the LPS-mediated inflammatory response [61]. 2-linoleoyl glycerol has the potential to attenuate the allergic diseases [62]. Use of 2-palmitoyl glycerol and 2-linoleoyl glycerol exhibited synergistic effect in the binding of 2-arachidonoyl glycerol for binding to the cannabinoid receptor 2 (CB-2) thus enhancing anti-nociceptive activities [63]. HPLC-DAD analysis of FXM indicated the existence of rutin and caffeic acid (data not shown). Oral administration of rutin significantly reduced the carrageenan induced paw edema in rat and also the migration of neutrophils [64]. Administration of rutin reduced the paw edema volume in adjuvant-carrageenan-induced inflammation in rats [65]. Palmitic acid induced inflammation on cultured macrophages and development of fatty liver in high fat fed diet in mice was suppressed with rutin treatment [66]. Caffeic acid reduces the acute immune and inflammatory response [67]. Anti-inflammatory and anti-coagulatory activities of caffeic have also been reported [68]. Studies suggest that caffeic acid also possesses antitumor abilities [69].

## Conclusion

On the basis of the results obtained in this study it can be speculated that plant has the ability to inhibit the LPS, TNF-α and carrageenan induced stimulatory responses, acetic acid and hot plate induced pain responses. The chloroform fraction (FXC) exhibited significant anti-inflammatory activities during in vitro and in vivo studies suggesting its therapeutic importance in inflammation related disorders.

## Abbreviations

COX, cyclooxygenase; FXA, *Fraxinus xanthoxyloides* residual aqueous fraction of methanol extract of leaves; FXB, *Fraxinus xanthoxyloides* n-butanol fraction of methanol extract of leaves; FXC, *Fraxinus xanthoxyloides* chloroform fraction of methanol extract of leaves; FXE, *Fraxinus xanthoxyloides* ethyl acetate fraction of methanol extract of leaves; FXH, *Fraxinus xanthoxyloides* n-hexane fraction of methanol extract of leaves; FXM, *Fraxinus xanthoxyloides* methanol extract of leaves; IL, interleukins; iNOS, indicible nitric oxide synthase; LPS, lipopolysaccharides; NFkB, necrosis factor kappa B; NO, nitric oxide; PG, prostaglandin; TNF-α, tumor necrosis factor -alpha
